# Pediatric C-spine Clearance by CT: A Retrospective Cohort Study

**DOI:** 10.7759/cureus.67832

**Published:** 2024-08-26

**Authors:** Anthony J Duncan, Mentor Ahmeti

**Affiliations:** 1 General Surgery, University of North Dakota School of Medicine and Health Sciences, Grand Forks, USA; 2 Department of Surgery, Sanford Medical Center, Fargo, USA

**Keywords:** pediatric trauma, cervical spine ct, cervical spine trauma, cervical collar, cervical spine clearance

## Abstract

Background: Pediatric cervical spinal injury (CSI) remains a significant concern following blunt trauma, with mortality rates as high as 48%. Current protocols involve cervical immobilization and clearance through multidetector computed tomography (MDCT) scans, followed by magnetic resonance imaging (MRI) or clinical examination. However, prolonged collar use poses risks, necessitating timely clearance. This study assessed the efficacy of MDCT in pediatric CSI clearance.

Methods: A retrospective cohort study, spanning January 2019 to January 2023, included pediatric patients under 18 undergoing cervical CT scans.

Results: MDCT sensitivity was evaluated, with 13.8% positive scans, detecting clinically significant injuries. MRI identified no additional injuries, affirming MDCT reliability. The average clearance time was 24.9 hours, impacting hospitalization durations. Mortality unrelated to CSI was excluded.

Conclusion: These results align with recent studies advocating cervical collar removal based on negative MDCT, emphasizing its potential to decrease the time that patients remain in C-collars and expedite hospital courses, including therapy and discharge. The study encourages consideration of MDCT-based protocols for timely pediatric CSI clearance, promoting patient care efficiency and informed medical decision-making.

## Introduction

Trauma remains a leading cause of morbidity and mortality in pediatric patients, with approximately 1% experiencing cervical spinal injury (CSI) following blunt trauma [1-4]. CSI can lead to significant mortality, which has been estimated as high as 48% [5]. Similar to adults, pediatric patients suspected of cervical injury undergo placement in a cervical collar, placement on a spine board with limited cervical movement until the spine is cleared. In the case of adults, evidence-based guidelines from the Eastern Association for Surgery and Trauma support the removal of cervical collars in blunt trauma cases with obtunded patients who have a normal multidetector computed tomography (MDCT) scan [6]. This recommendation was further validated by a prospective study by the Western Trauma Association [7].

By contrast, the current standard for pediatric patients requires clearance through a normal MDCT scan, followed by either a normal cervical MRI or a normal clinical examination [8]. While the significance of cervical spine clearance in children is paramount, it is essential to acknowledge that no medical intervention is without risks. Prolonged use of cervical collars in pediatric patients may lead to iatrogenic pressure ulcers, increased difficulty in intubation, elevated risk of aspiration, and heightened intracranial pressures [9-13]. Therefore, minimizing the time to cervical spine clearance is crucial when feasible. MDCT scans have demonstrated a high sensitivity of 81-100% in pediatric patients over time [14-18]. Moreover, when pediatric trauma patients present with concerns about cervical injury, they typically undergo a cranial MDCT, facilitating an extension of the scan for cervical imaging.

This study seeks to assess the role of MDCT in pediatric patient clearance, aiming to refine local protocols for pediatric cervical spine clearance and contribute valuable insights to the broader discourse on best practices within the medical community.

## Materials and methods

This study was assessed and deemed exempt by our institutional review board (IRB STUDY00003348). We conducted a retrospective cohort study at a single-center adult level I/pediatric level II trauma center in Fargo, North Dakota, from January 2019 to January 2023. The inclusion criteria encompassed all patients under 18 years old who presented with trauma and underwent a cervical CT scan. Patients were excluded if ≥ 18 years old, had a penetrating neck injury, had in-hospital mortality due to a cause not related to their cervical spine, had C-spine cleared prior to transfer to our institution or transferred to another institution without clearance of C-spine, or if the C-collar was removed for ICP management.

Upon identifying eligible patients, a manual chart review was conducted. Study data were collected and managed using REDCap electronic data capture tools [19,20]. The collected data included age, race, gender, presentation date and time, Injury Severity Score (ISS), initial Glasgow Coma Scale (GCS), mechanism of injury (MOI), presence of C-spine tenderness, the presence of a cervical collar on presentation, consultation with neurosurgery and subsequent recommendations, MDCT results, whether a patient had an MRI and the subsequent results, time to clearance of the cervical spine, length of hospital stay, and discharge location.

During the study period, MDCT scans were obtained following our institutional guidelines, which, during the study period, included patients whose GCS was less than 15 and whose clinical exam could not be obtained, those with altered mental status, or those with a high-risk mechanism (i.e., a motor vehicle accident with ejection). Follow-up MRIs were then obtained for patients under 18 years old with neurological findings, cervical spine tenderness with a negative CT scan, and positive CT scans with neurosurgical requests for additional imaging.

Statistical analysis was performed using R (R: A language and environment for statistical computing, R Foundation for Statistical Computing, Vienna, Austria), with basic demographic statistics conducted [21]. Our paper was written to adhere to the Strengthening the Reporting of Observational Studies in Epidemiology (STROBE) reporting guidelines (Appendix), which is included as part of the appendix content for this article.

## Results

Demographic information

We identified 289 patients that met the inclusion criteria in our database. Our study population had a mean age of 11 years old (standard deviation (SD) = 5.8) with a distribution of 17 (5.9%) patients less than one year old, 50 (17.3%) patients between one and four years old, 50 (17.3%) patients between five and nine years old, 70 (24.2%) patients between 10 and 14 years old, and 102 (35.3%) patients between 15 and 17 years old. Among them, 168 (57.9%) were male. In terms of race, White was the most common, with 198 (68.6%) patients, followed by American Indian or Alaska Native (65, 21.4%), Black or African American (18, 6.2%), Asian (4, 1.3%), and unknown (4, 1.3%) (Table 1).

**Table 1:** Demographics information and presentation ^1^mean (SD); ^2^n (%)

Age^1 ^ (n = 289)	10.9 (5.8)
<1year old^2^	17 (5.9)
1-4 years old^2^	50 (17.3)
5-9 years old^2^	50 (17.3)
10-14 years old^2^	70 (24.2)
14-17 years old^2^	102 (35.3)
Race (n = 289)	
White^2^	198 (68.6)
American Indian/Alaska Native^2^	65 (21.4)
Black or African American^2^	18 (6.2)
Asian^2^	4 (1.3)
Unknown^2^	4 (1.3)
Gender (n=289)	
Male^2^	168 (57.9)
Female^2^	121(42.1)
Mechanism of Injury (n = 289)	
Vehicle/MVC^2^	92 (32.1)
Fall/drops^2^	56 (19.3)
ATV^2^	29 (10.0)
Pedestrian struck by car^2^	20 (6.9)
Assault/abuse^2^	15 (5.2)
Ski/snow^2^	14 (4.8)
Sport related^2^	8 (2.8)
Other/unknown^2^	57 (20.0)
Initial presentation (n = 289)	
C-collar on Presentation	
Yes^2^	191 (66.1)
No^2^	98 (33.9)
ISS mean^1^	11.2 (10.6)
GCS mean^1^	13.3 (3.8)

Initial presentation

The most common mechanism of injury was a motor vehicle collision in 92 (32.1%) patients, with falls or drops ranked second in 56 (19.3%) patients. The majority of patients who underwent cervical imaging presented with a C-collar in place, accounting for 191 (66.1%) patients. Neurosurgery was consulted on presentation in 98 (33.9%) patients for either cervical or intra-cranial concerns. In all cases where neurosurgery was consulted, they determined clinical C-spine clearance (Table 1).

Imaging

All patients underwent cervical MDCT, of which 40 (13.8%) were positive. Of the positive CT scans, 35 represented injuries that needed intervention. Seventeen (5.9%) patients required surgery, and 13 (4.5%) required the use of a rigid collar with the remaining patients only needing soft collars for comfort (n = 5, 1.7%). Of the 40 positive MDCT scans, 14 (4.8%) patients received an MRI, with no instances of MRI showing additional injuries that were not present on the CT scan. The remaining 26 (9.0%) patients did not get an MRI as it would not change their clinical course.

**Figure 1:**
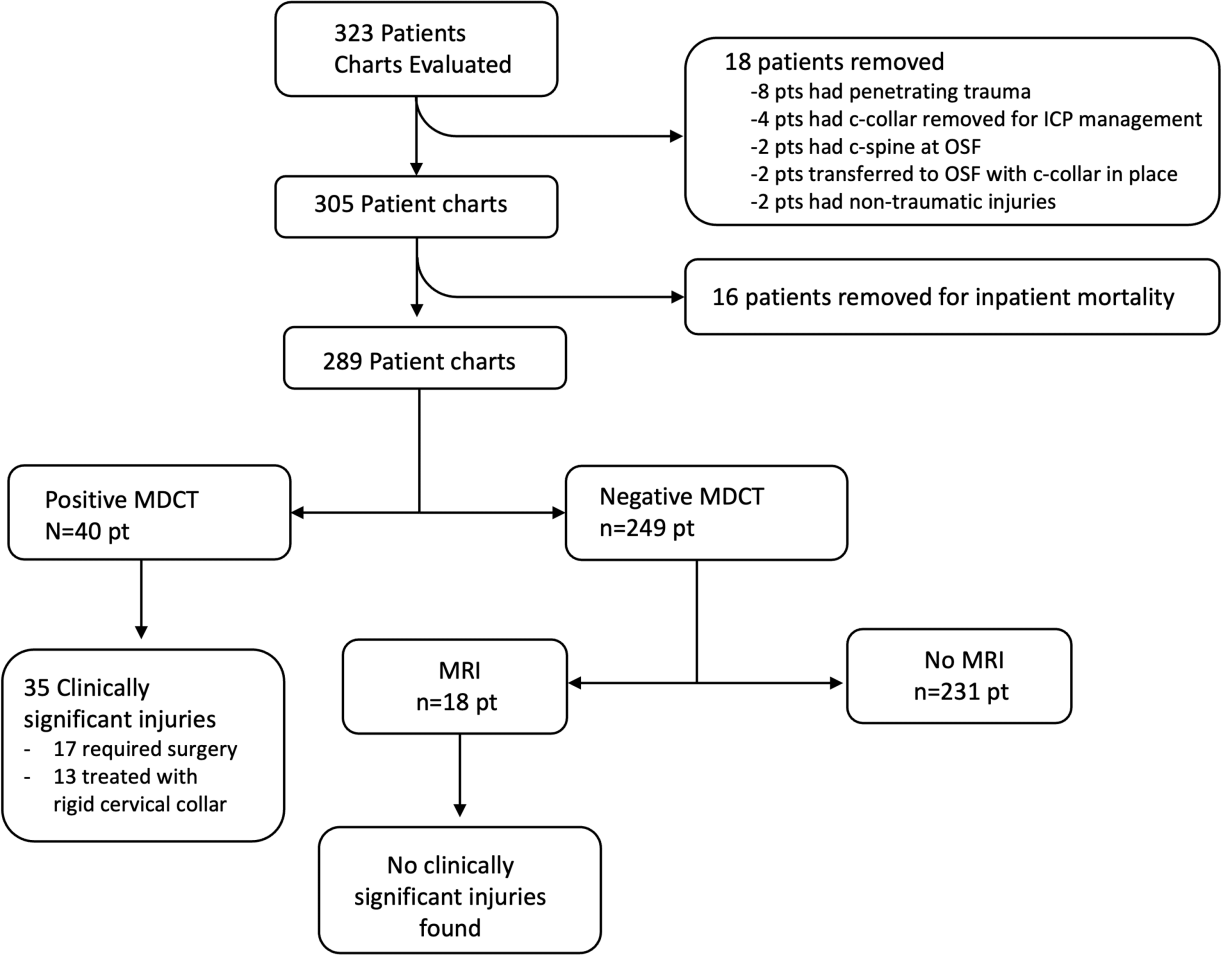
Patient imaging distribution

Among the 249 patients with negative CT scans, 18 (6.2%) patients had an MRI of the cervical spine. MRI was obtained in five (1.7%) of these patients due to C-spine tenderness on examination, five (1.7%) patients per neurosurgical consultation secondary to mechanism of injury, and eight (2.8%) patients for clearance of the patient’s cervical spine while intubated. One patient had a mild signal abnormality, one had a small amount of paravertebral edema, and three patients' MRI results were negative. All five patients were discharged with soft cervical collars for comfort for two weeks. Eight of the MRIs were ordered for cervical clearance in intubated patients, all of which were negative (Figure 1).

C-spine clearance and discharge

Of the 249 patients who had a negative CT scan, 191 (66.1%) patients presented with cervical collars, and 37 (12.8%) patients were discharged with C-collars and neurosurgical follow-up. The remaining 154 (53.3%) patients had their cervical spine cleared clinically during their hospitalization. No incidence of skin breakdown or direct complications from the cervical collar was found within the cohort. No patients were found to have delayed bony or spinal cord injuries. For all patients with cervical collars in place, it took an average of 24.9 hours (SD 67.3; max 734 hours) to clear the cervical spine. On average, the patients were discharged at a mean of 5.4 days (SD = 10.7). Of the original 289 patients, the majority were discharged home (88.2%, n = 255). 7.2% (n = 21) went to short-term rehab, 1.7% (n = 5) were discharged with home health, 1.4% (n = 4) were discharged to hospice, 0.7% (n = 2) were discharged to inpatient psychiatry, and 0.7% (n = 2) left AMA. Patients with mortality not related to cervical injury were not included in the study, and there was no mortality associated with cervical injury in the patient population (Table 2).

**Table 2:** C-spine clearance and discharge disposition ^1^n (% of total); ^2^mean (SD)

Cervical spine clearance	
Patients that had CT C-spine^1^	249 (86.1l)
Presented to ED with C-collar^1^	191 (66.0)
Cervical spines cleared during hospitalization^1^	154 (53.2)
Time to spine clearance (hours)^2^	24.9 (67.3)
Hospital length of stay (days)^2^	5.4 (10.7)
Discharge features	
Discharged with cervical collar^1^	37 (12.8)
Discharged home^1^	255 (88.2)
Discharged to short term rehab^1^	21 (7.2)
Discharged home with home health^1^	5 (1.4)
Discharged to hospice^1^	4 (1.4)
Discharged to inpatient psychiatry^1^	2 (0.7)
Left AMA^1^	2 (0.7)

Outpatient C-spine clearance

Thirty-seven patients were discharged with C-collars, which also included those who had operative interventions by Neurosurgery. Twenty-three were discharged with rigid/hard collars, and 14 were discharged with a soft collar. The average time until neurosurgical clearance as an outpatient was 59.4 days (SD = 44.3, Min: 16, Max: 219). No complications of the collar were appreciated within this group.

## Discussion

This study looked at the utility of MDCT in identifying clinically relevant injuries in pediatric patients who underwent blunt trauma. The demographic breakdown of our cohort exhibited a diverse age distribution, with a slight male predominance. The racial composition was predominantly white, consistent with other studies and reflective of broader population demographics [16,22,23]. Our study had a larger representation of American Indian and Alaska Native (AIAN) individuals compared to national data, given our unique and diverse catchment area. Motor vehicle collisions emerged as the predominant mechanism of injury, aligning with existing literature on pediatric trauma [23].

The patient population was limited to those who had MDCT scans, conducted in accordance with institutional guidelines. This allowed us to evaluate the sensitivity of MDCT within our blunt trauma patient population. Among the patients who received MRI after MDCT, we found no additional significant injuries. Five patients who received an MRI had a negative CT but reported cervical spine tenderness. None of these patients required surgical intervention, and none had significant injuries. They were discharged home with a soft collar for comfort.

Clearing the cervical spine on an obtunded adult patient has been scientifically validated by the Eastern Trauma Association 2015 Guidelines [6]. However, this practice has not yet been extended to the pediatric patient population, leaving pediatric C-spine clearance challenging. Furthermore, it is difficult to easily extrapolate data from adult patients to apply to pediatric patients [24]. The clearance of the C-spine is challenged by evidence showing that Canadian C-spine Rules and Nexus Criteria do not perform well in younger pediatric patients [25,26]. Limiting radiation exposure in pediatric patients also remains fundamental for appropriate medical management [27].

Patients who have already received an MDCT of the cervical spine and a clinical exam that is unable to be obtained can be cleared without further imaging or clinical examination. These findings further support the study performed by Russell et al., who similarly found an MDCT sensitivity of 100% in their retrospective study of 4,477 pediatric trauma patients [16]. Prior to their work, two other studies also supported the ability to clear the cervical spine with a normal MDCT [24,28]. Derderian et al. showed in their 221-patient study that no clinically significant cervical spinal injuries were found if patients had a normal CT [29]. Patients might also benefit from CT C-spine guidelines similar to those by Douglas et al., which decreased the number of full C-spine CTs. Within our institution, we had previously implemented a protocol similar to Douglas et al.; however, shortly after, it had missed a C7 injury, thus discontinuing the limited C-spine scan.

Limitations

While our study contributes valuable insights into the sensitivity of MDCT in pediatric cervical spine clearance, several limitations should be acknowledged. First, the retrospective nature of our study introduces inherent biases and limitations associated with retrospective data collection and analysis, including the potential for selection bias and incomplete data capture. In addition, the study was conducted at a single-center trauma center, which may limit the generalizability of our findings to other settings with different patient populations, resources, and protocols. Furthermore, our sample size is limited in comparison to other studies. Lastly, our study did not assess long-term outcomes or complications associated with cervical spine clearance and the use of cervical collars in pediatric patients. 

## Conclusions

This retrospective review of blunt pediatric injuries shows that no significant injuries were missed by MDCT. The absence of additional injuries on MRI scans confirms the reliability of MDCT in detecting cervical spine injuries in the pediatric population. This is further evidence in line with recent studies, supporting the removal of cervical collars based on negative MDCT in this population. Furthermore, earlier clearance could aid in the patient's progression through their hospital course with physical therapy and earlier discharges.

## Appendices

**Table 3:** Strengthening the Reporting of Observational Studies in Epidemiology (STROBE) checklist for cohort studies

	Item No.	Recommendation	Page No.
Title and abstract	1	(a) Indicate the study’s design with a commonly used term in the title or the abstract	Title page
		(b) Provide in the abstract an informative and balanced summary of what was done and what was found	Abstract
Introduction			
Background/rationale	2	Explain the scientific background and rationale for the investigation being reported	1
Objectives	3	State specific objectives, including any prespecified hypotheses	1-2
Methods			
Study design	4	Present key elements of study design early in the paper	2
Setting	5	Describe the setting, locations, and relevant dates, including periods of recruitment, exposure, follow-up, and data collection	2
Participants	6	(a) Give the eligibility criteria, and the sources and methods of selection of participants. Describe methods of follow-up	2
		(b) For matched studies, give matching criteria and number of exposed and unexposed	N/A
Variables	7	Clearly define all outcomes, exposures, predictors, potential confounders, and effect modifiers. Give diagnostic criteria, if applicable	2
Data sources/ measurement	8*	For each variable of interest, give sources of data and details of methods of assessment (measurement). Describe comparability of assessment methods if there is more than one group	2
Bias	9	Describe any efforts to address potential sources of bias	2
Study size	10	Explain how the study size was arrived at	3
Quantitative variables	11	Explain how quantitative variables were handled in the analyses. If applicable, describe which groupings were chosen and why	N/A
Statistical methods	12	(a) Describe all statistical methods, including those used to control for confounding	2
		(b) Describe any methods used to examine subgroups and interactions	N/A
		(c) Explain how missing data were addressed	N/A
		(d) If applicable, explain how loss to follow-up was addressed	N/A
		(e) Describe any sensitivity analyses	N/A
Results			
Participants	13*	(a) Report numbers of individuals at each stage of study—eg numbers potentially eligible, examined for eligibility, confirmed eligible, included in the study, completing follow-up, and analysed	3
		(b) Give reasons for non-participation at each stage	3
		(c) Consider use of a flow diagram	Figure 1
Descriptive data	14*	(a) Give characteristics of study participants (eg demographic, clinical, social) and information on exposures and potential confounders	3
		(b) Indicate number of participants with missing data for each variable of interest	N/A
		(c) Summarise follow-up time (eg, average and total amount)	N/A
Outcome data	15*	Report numbers of outcome events or summary measures over time	3-4
Main results	16	(a) Give unadjusted estimates and, if applicable, confounder-adjusted estimates and their precision (eg, 95% confidence interval). Make clear which confounders were adjusted for and why they were included	4-5
		(b) Report category boundaries when continuous variables were categorized	N/A
		(c) If relevant, consider translating estimates of relative risk into absolute risk for a meaningful time period	N/A
Other analyses	17	Report other analyses done—eg analyses of subgroups and interactions, and sensitivity analyses	N/A
Discussion			
Key results	18	Summarise key results with reference to study objectives	5
Limitations	19	Discuss limitations of the study, taking into account sources of potential bias or imprecision. Discuss both direction and magnitude of any potential bias	6
Interpretation	20	Give a cautious overall interpretation of results considering objectives, limitations, multiplicity of analyses, results from similar studies, and other relevant evidence	6
Generalisability	21	Discuss the generalisability (external validity) of the study results	6
Other information			
Funding	22	Give the source of funding and the role of the funders for the present study and, if applicable, for the original study on which the present article is based	N/A
